# Touch-Point Detection Using Thermal Video With Applications to Prevent Indirect Virus Spread

**DOI:** 10.1109/JTEHM.2021.3083098

**Published:** 2021-05-24

**Authors:** Guangshen Ma, Weston Ross, Matthew Tucker, Po-Chun Hsu, Daniel M. Buckland, Patrick J. Codd

**Affiliations:** 1 Department of Mechanical Engineering and Materials ScienceDuke University 3065 Durham NC 27705 USA; 2 Department of NeurosurgeryDuke University 3065 Durham NC 27710 USA; 3 Division of Emergency MedicineDepartment of SurgeryDuke University 3065 Durham NC 27707 USA

**Keywords:** Image processing, health and safety, photothermal effects, sanitary engineering

## Abstract

Viral and bacterial pathogens can be transmitted through direct contact with contaminated surfaces. Efficient decontamination of contaminated surfaces could lead to decreased disease transmission, if optimized methods for detecting contaminated surfaces can be developed. Here we describe such a method whereby thermal tracking technology is utilized to detect thermal signatures incurred by surfaces through direct contact. This is applicable in public places to assist with targeted sanitation and cleaning efforts to potentially reduce chance of disease transmission. In this study, we refer to the touched region of the surface as a “touch-point” and examine how the touch-point regions can be automatically localized with a computer vision pipeline of a thermal image sequence. The pipeline mainly comprises two components: a single-frame and a multi-frame analysis. The single-frame analysis consists of a Background subtraction method for image pre-processing and a U-net deep learning model for segmenting the touch-point regions. The multi-frame analysis performs a summation of the outputs from the single-frame analysis and creates a cumulative map of touch-points. Results show that the touch-point detection pipeline can achieve 75.0% precision and 81.5% F1-score for the testing experiments of predicting the touch-point regions. This preliminary study shows potential applications of preventing indirect pathogen spread in public spaces and improving the efficiency of cleaning sanitation.

## I. Introduction

Human infections can be caused by viruses and bacterial pathogens that are transmitted through contaminates surfaces [1], [2]. Previous research has shown that the virus particles can stay at the surfaces for a relatively sufficient time and this indicates the surface can play important role for viral spreading [3], [4]. For example, in the global pandemic of coronavirus disease 2019 (COVID-19), the virus is most commonly transmitted through respiratory droplets exchanged between people, but a secondary means of transmission for the virus is through contact with contaminated surfaces [5]. As with other transmissible diseases, respiratory droplets emitted from a contagious carrier can land on surfaces that are touched by other people and can lead to infection through the eyes, mouth, or respiratory system. Evidence shows that the virus can live on the surface of skin for about 9 hours [6], about 5 times longer than the common influenza virus, and can exist for days on common surfaces [7]. In public and shared spaces, where surfaces are frequently touched by multiple people, risk of transmission can be reduced with sufficient surface sanitation [8]. However, exhaustive cleaning efforts are not always attainable due to the cost, time, and workforce limitations. The ability to determine which surfaces have been touched could dramatically improve the efficiency of targeted touch-point cleaning efforts and improve the completeness of sanitation in public spaces (e.g classroom, office, airports, clinical settings) to reduce the spread of viruses. This paper addresses this challenge through computer vision algorithms with thermal imaging to detect and track touch-points.

### A. Thermal Signatures of Touch-Points

**FIGURE 1.**
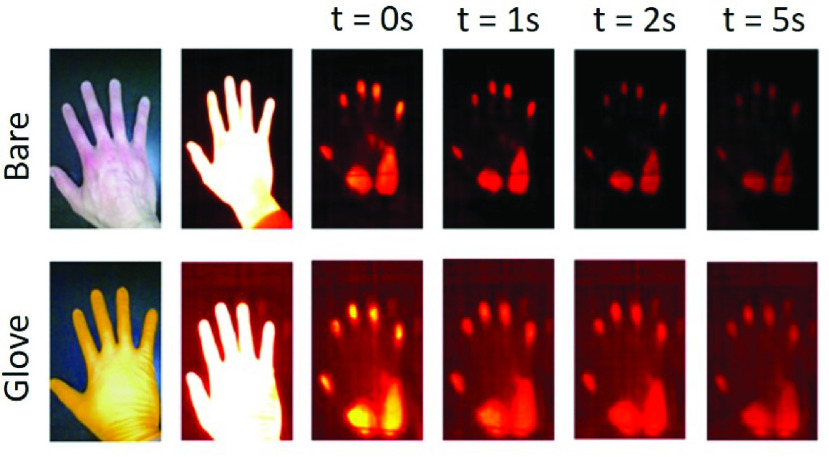
Touch-points at the surface with two touching patterns: bare touching and wearing gloves. Touch-points are detected through their thermal signatures that would decay over time.

A salient feature of the touch-point is the thermal signature, which is an indication of heat transfer through contacted surfaces. When a person touches a surface (e.g. a table), heat is transmitted into the surface leaving behind a thermal signature. This process is a result of conductive heat transfer, which is affected by the temperature difference among the person, the surface and the surrounding air, the material thermal properties and the time of contact [9]. The resulting thermal signature can be seen as a ‘hot spot’ in a thermal camera image. Over time, the heat dissipates into the surface or surrounding air and the thermal signature disappears. For regular surface materials such as wood, metal, fabric and ceramics, the thermal signatures can leave at the surface for several seconds (Fig. 1).

The thermal image consists of an array of pixels encoding relative temperature, with greater pixel intensity denoting a higher temperature value. However, a single image does not contain temporal information, which can be used to distinguish between transient thermal signals (e.g. touch-points) and persistent thermal signals. A video encodes both the spatial and temporal information of the contacted surface and hence it is capable of detecting a touch-point with greater accuracy.

Analysis of the thermal video is similar to that of the standard digital video, except there is only one channel of information per pixel, i.e. the pixel intensity, much like a grayscale image. The touch-point detection requires both spatial (intraframe) and temporal (interframe) feature extraction. In particular, we are interested in extracting the feature of the residual thermal heat (or thermal signature) that remains after a surface has been touched, assuming that the surface is cooler than the person touching it and that the touch imparts heat to the surface. Heat signature has been employed to monitor hand gestures on interactive surfaces [10] and to track which objects a dementia patient has recently touched in the absence of their caregiver [11]. Similar to these studies, this work seeks to detect thermal signatures from thermal videos.

### B. Thermal Video Processing Techniques

In a thermal video, the human subject and the touch-points are both thermal signatures that are transient and may be moving, compared to the background image which is relatively stationary and does not change temperature quickly. The background image shows a very uniform pixel intensity over time, since most of the objects have a temperature close to the environmental temperature in a room. Therefore, one method of separating a signal of interest (i.e. touch-point, human subject and other background objects) is to perform Background subtraction [12]. Background subtraction aims to detect touch-point regions based on the difference between the current and the reference image, which has been widely used in visual analysis for human activities [13], [14] and segmenting moving objects for video analysis [15], [16]. More importantly, Background subtraction is capable of segmenting the pixel regions with different intensity from the background image, and hence the objects that are moved or touched can be identified.

In this study, the main task is to distinguish between the foreground (human subject) and ultimately any changes to the background (e.g. touch-points) that occur, because the human subject shows similar temperature distributions compared with the touch-point. However, it is difficult to model this process, since the physical interaction for a single contact is related to various factors such as the time of contact, surface temperature, ambient temperature and etc. This complex physical interaction makes it difficult to design a precise feature extractor and a robust classifier for touch-point prediction. Therefore, we consider the detection of touch-points as a semantic segmentation problem and employ deep learning tools for this computer vision task.

### C. Deep Learning in Thermal Image Semantic Segmentation

Classification of the touch-points and human subjects can be modelled as a semantic segmentation problem, which aims to assign a label to every pixel of an image as an indication of a specific category. Semantic segmentation is a well-studied problem and researchers have proposed various deep learning model architectures in this field [17], [18]. Three of the most powerful networks for semantic segmentation are U-net [19], FCN [20] and SegNet [21], which leverage the benefits of convolutional neural networks (CNN) to formulate the models. For semantic segmentation, FCN and SegNet are usually initialized with a pre-trained model (e.g. Vgg16 model [22]) to speed up the training process, which can efficiently extract the image features and adapt to new computer vision tasks with less training data [23]. However, the task of touch-point detection is very different from these pre-trained models. If the FCN or SegNet models were to be trained from scratch, the model would still require a large amount of data, while the proposed study can only provide a small training dataset since it is a new dataset, namely thermal videos with touch-point events. Compared with FCN and SegNet, U-net has the advantages of high computational efficiency, trainable with small datasets, and fewer model parameters.

In summary, most of the related studies focused on the use of thermal signature for various applications such as surface interaction in human computer interface [10], material detection through thermal signatures [24], [25] and touch gesture detection [26]–[28]. None of these studies addressed the task of using a thermal camera for touch-point region detection. To the authors’ knowledge, this is the first study to achieve touch-point detection by using thermal videos.

This paper is organized as follows. Section II presents a computer vision pipeline that comprises a single-frame analysis of using U-net for touch-point segments and a multi-frame analysis of creating a cumulative probability map of the touched regions; Section III shows the experimental results; Section IV analyzes the performance of the proposed method and discusses the applications of this technology.

## II. Methods

### A. Terminology and Assumptions

#### 1) Terminology

The following terminologies are defined below for clarity throughout the paper:

• *Human-subject:* A person in the frame of the thermal image and the source of the heat for the touch-points. The Human subject is usually moving in the thermal video.
• *Touch-point:* A connected pixel region that exhibits a higher pixel intensity (i.e. temperature) compared with the background object at the same pixel locations. The touch-point is a transient and time-decaying target.
• *Background:* The pixel regions in the frame different from the “Touch-point” and “human-subject” and generally refer to all surfaces in the environment that are unchanging, both thermally and spatially.

#### 2) Assumptions

Touch-point prediction is a complex process that is affected by various factors such as moving objects in the image, temperature difference and a variety of thermal targets. Hence, we make the following assumptions and simplifications for this study:

• The background objects (other than touch-points and human-subjects) are static and should not be moved.
• The human subject is warmer than the room temperature and the touched surfaces. The heat can be imparted to the surface during contact, creating a touch-point of higher intensity in the thermal image. This represents the vast majority of use cases, particularly for sanitation related efforts, since surfaces in a room are usually at ambient room temperature.

### B. Data Collection

**FIGURE 2.**
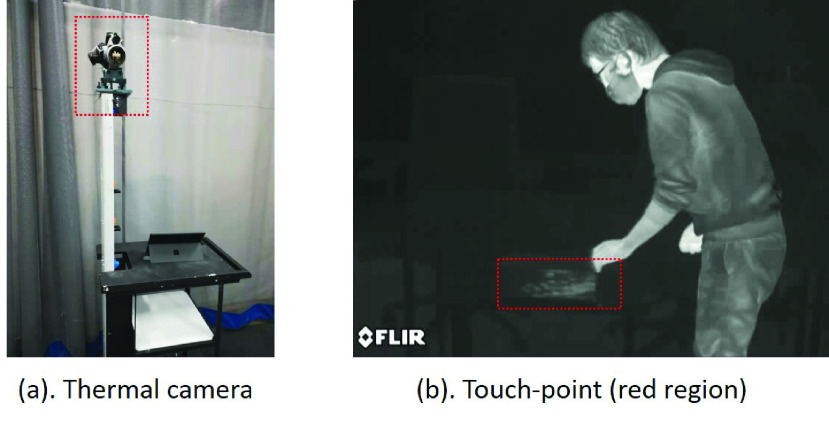
(a): Thermal camera for data collection. (b): Thermal image includes a touch-point (red region) and a human-subject.

In preparation for algorithm development, we gathered thermal videos from several representative environments and scenarios in which touch-points are commonly generated. The data was collected in four locations: a shared 3D printing studio, a common office room, a conference room, and a laboratory space configured to mimic a clinical exam room. This ensured that the videos contained touch-point events for a variety of lighting conditions and surface material types. The video was collected with the FLIR SC640 thermal camera (FLIR Systems, Arlingong, VA) that has an IR image resolution of $640 \times 480$ pixels at 30 frames per second (fps) and a thermal sensitivity of $30 mK$ at room temperature (Fig. 2 (a)). This camera setting can sufficiently record the change of thermal signatures and automatically adjust the pixel intensity to match the temperature range of 20 to 35 degrees Celsius.

A total of 30 videos, each about 60 seconds long, were collected and each contained about 1 to 5 touch-point events. The recorded videos were converted to image sequences and re-sampled down to 15 frames per second for processing. In each video, the human subject intentionally created several touch-points on objects in the scene. The touch-points were clearly visible immediately following the touch event and some reference images were manually selected as representations of the touch-point regions in the video. The criteria for choosing the reference image was that it contained at least one touch-point region and one human subject, and that the human subject was not occluding the touch-point. This standard ensured that the features of the touch-points and the human subject were included in the reference images. In summary, we split the 30 videos into two datasets based on the ratio of 70/30:

• The training dataset included 21 thermal videos. Each video was first converted to an image sequence. The reference images were selected from these images to formulate a dataset for U-net model training and validation. In addition, the evaluation of the touch-point detection pipeline was also conducted in this dataset and the input was the complete image sequence for each video.
• The testing dataset included 9 thermal videos. This dataset excluded from model training and only used for the evaluation of the touch-point detection pipeline performance after training.

### C. Image Preprocessing

**FIGURE 3.**
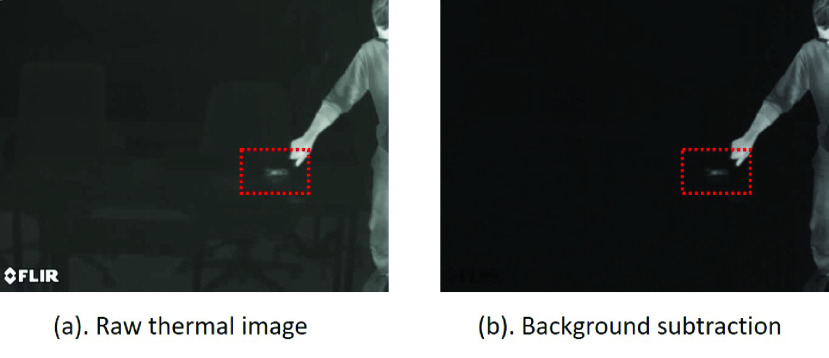
(a): The image before Background subtraction. (b): the image after Background subtraction. The red region is noting the touch-point feature. Except for the touch-point and human-subject, the other pixel regions show very low pixel intensity after Background subtraction.

The image preprocessing steps are applied to all the images from the thermal videos. Each frame is a grayscale image with pixel intensities ranging between 0 and 255. The videos were collected in a room temperature around 25 degree Celsius and each pixel intensity was scaled to this temperature range. In order to amplify the signals of interest (i.e. touch-points and human-subject), each frame was normalized by pixel-wise subtraction of the background image (i.e. Background subtraction), which was captured initially when no human-subject occurs in the scene. Fig. 3 illustrates the difference of the raw image and the image after Background subtraction.

Background subtraction is capable of filtering the pixel regions with low intensity changes, thus highlighting the differences, of which a touch-point is an important difference to detect. The pixel regions being touched or blocked by a human-subject will be highlighted in the frame, as shown in Fig. 3. It is noted that the variations of background image will affect the touch-point prediction when some background objects are moving periodically. This is because the “moving object” will cause very unpredictable changes of pixel intensity, and makes it difficult for touch-point detection. Additionally, if a background image is captured when a human object is in the scene, the variation caused by human movement will inevitably affect the touch-point prediction. However, these problems require a more complex design of the computer vision method and are out of scope of this study. As proof of concept, we have made an assumption that only static background objects are considered.

### D. Touch-Point and Human-Subject Features

After preprocessing each frame, the next step is to identify the signals of interest between touch-points and human subjects. The difference of touch-point and human subject can be summarized into three categories: pixel intensity, shape of the tracked object and temporal intensity changes. Since different parts in the human body can show various temperatures ranged from 25 to 35 degrees Celsius, the pixel intensity between the touch-point and the human subject is overlapped and thus cannot provide valuable information for segmentation. The main difference between the touch-point and human subject signals is the spatial relation between pixels. The touch-point is usually a small pixel region while the human subject is a larger one. These features can be easily learned by the convolutional layers in the U-net model.

**FIGURE 4.**
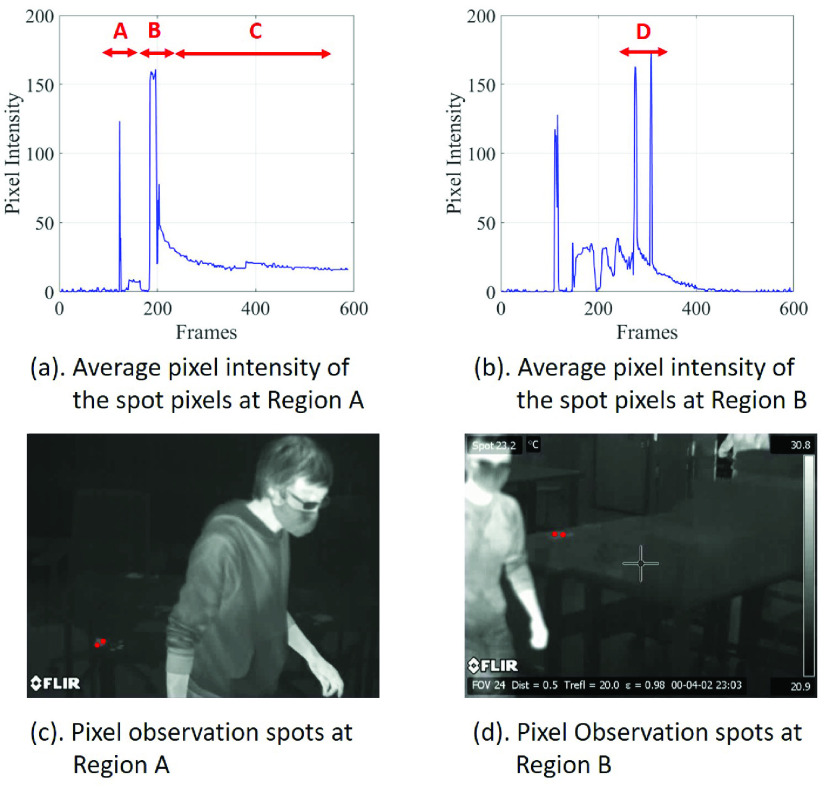
Average pixel intensity at the observation spots. Fig. (a) and Fig. (b) describe different touch-point events. A: Subject enters to the frame. B: Touch-point starting. C: Pixel intensity decays. D: Subject enters to the frame again and the decay of pixel intensity is interrupted. Fig. (c) and Fig. (d) show the corresponding observation spots (red dots) in Fig. (a) and Fig. (b), respectively.

There are usually three distinct periods or events by tracking temporal intensity of a single pixel in a thermal video. Fig. 4 shows the average pixel intensity of the observation spots in the same touch-point region. In Fig. 4 (a), Event A shows that a subject (with higher temperature) enters the frame and block the spot, resulting in the rise of pixel intensity; Event B shows that a subject touches surface and the pixel intensity increases; Event C shows that a subject leaves the frame and the pixel intensity begins to decrease, because the heat source (human-subject) leaves the surface and the surrounding air has lower temperature. The magnitude of the pixel intensity between event A and B are similar, which makes a simple threshold-based image segmentation difficult to be applied. Furthermore, the data between the $1^{st}$ to $100^{th}$ frames (in Fig. 4 (a)) demonstrates a non-touching period, where the pixel intensity remains at a stable value without temperature changes. Event C shows that even during the decay period after the touching behaviours, the pixel intensity is higher than the background pixel intensity. The thermal signature can still be visually detected in the thermal video for a relatively long time.

Another problem is that human subject moving in the thermal video will greatly affect the temporal feature. It is likely that the human subject will occupy a pixel position at the observation spot. The pixel intensity will first increase for a period of time and decay again. The effect of the human subject is usually unpredictable and a feasible solution is to use the semantic segmentation method to segment the human target in the image frame and highlight the touch-point regions. For example in Fig. 4 (b), Event D denotes a situation where the human subject accidentally moves close to the camera and blocks the view of the pixel spot, resulting in two intensity peaks. This unpredictable behavior will greatly change the temporal feature of the pixel intensity and makes it difficult to detect the touch-point by model-based methods.

In summary, the analysis of temporal pixel features indicates the complexity of the touch-point detection and the difficulty of using model-based methods, and this motivates the use of deep learning tools, such as the U-Net model, for touch-point region segmentation.

### E. Touch-Point Detection Pipeline

**FIGURE 5.**
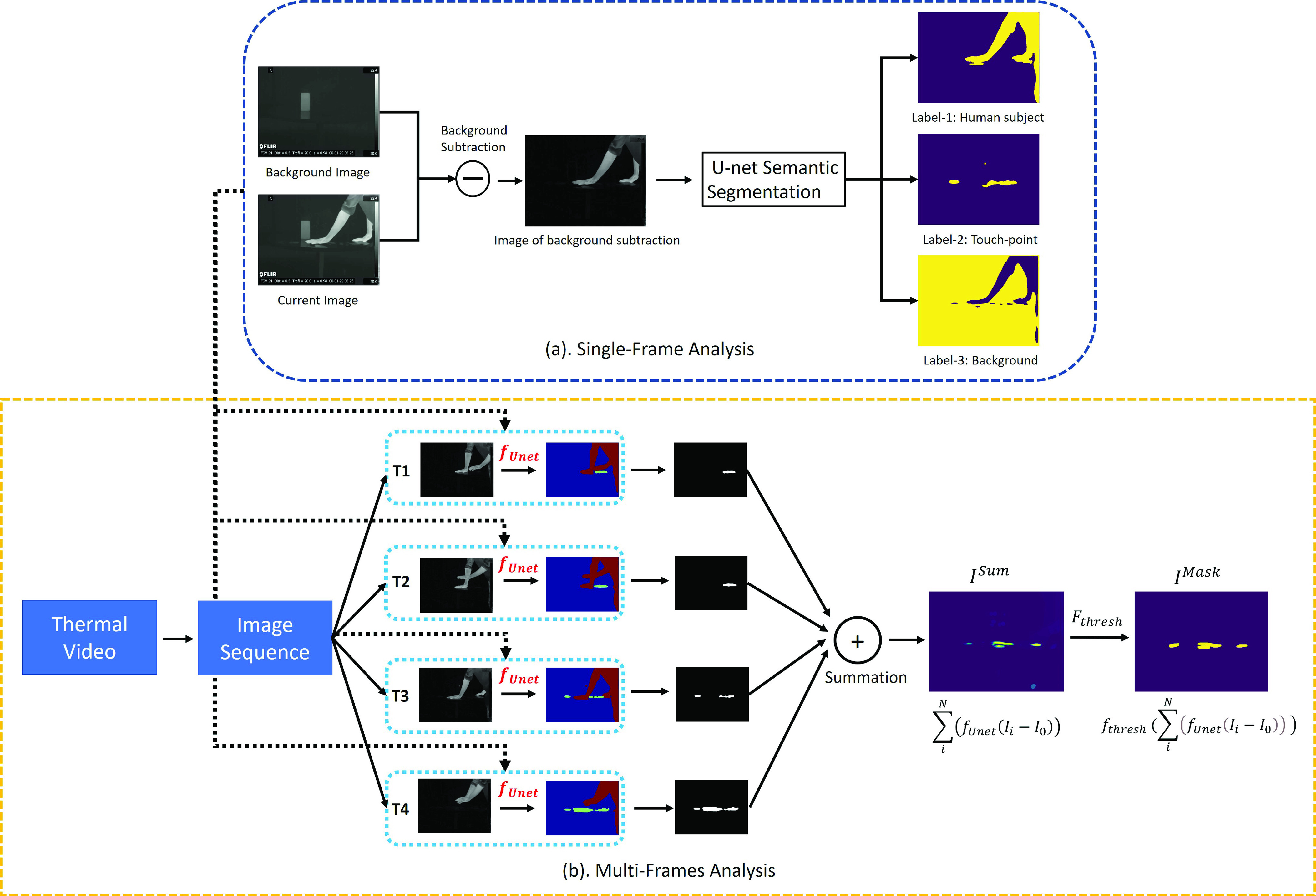
The touch-point detection pipeline. The blue box shows a single frame analysis using the U-net deep learning model. The U-net outputs are three segments (Human subject, Touch-point and Background). The yellow box shows the multi-frame analysis of summing the touch-point segments from the U-net outputs, which can formulate a cumulative probability map for touch-points prediction. $T_{1}$, $T_{2}$, $T_{3}$ and $T_{4}$ are the notation of different time steps in the video (not in order). “U-net semantic segmentation” (in Single-Frame analysis) refers to the generic deep learning model in [19].

This section describes a touch-point detection pipeline that consists of a single-frame and a multi-frame analysis. The single-frame analysis comprises a Background subtraction for preprocessing and a U-net deep learning framework for touch-point segmentation. U-net consists of an encoder-decoder architecture that can be used to capture image features and perform precise localization based on the convolutional operation [19]. Based on the unique features between touch-point and human subject signals, U-net is capable of distinguishing different pixel regions with various pixel intensity distributions and classify the segments appropriately. Once trained, it can identify the touch-point feature apart from the human subject in a single frame. For the multi-frame analysis, the outputs from the U-net based single frame analysis can be summed across the entire video image sequence to achieve our final touch-point prediction. The touch-point detection pipeline is summarized in Fig. 5.

Specifically, given an image sequence of N frames from a thermal video referred as $\overrightarrow {I} = \{ I_{1}, I_{2}, \cdots, I_{N} \}$, the goal is to train a function that maps $\overrightarrow {I}$ to an image mask $I^{mask}$ to show the pixel-wise location of the touch-points. The relation between $I_{i}$ and $I^{mask}$ can be represented as:

\begin{equation*} I^{mask} = f_{thresh} \left({\sum _{i}^{N} f_{Unet} (I_{i} - I_{0})}\right)\tag{1}\end{equation*}

where $f_{Unet}(I_{i} - I_{0})$ is the i-th U-net output with the touch-point semantic label after the Background subtraction between the current frame $I_{i}$ and the background image $I_{0}$. $\sum _{i}^{N} f_{Unet} (I_{i} - I_{0})$ is the summation of the touch-point segments from each U-net output. This can be regarded as a “confidence” score of a touch-point label or a duration time for a single spot observation. $f_{thresh}$ is used to filter the pixels with small intensity values, i.e. less likelihood of being a touch-point. The threshold of $f_{thresh}$ is set as 50 pixels for precise detection and this shows that the thermal signature of the touch-point should stay at the surface at least 3.3 seconds. Finally, $I^{mask}$ is the final output and each pixel location denotes the binary classification of the touch-point.

#### 1) Labelled Dataset for U-Net

**FIGURE 6.**
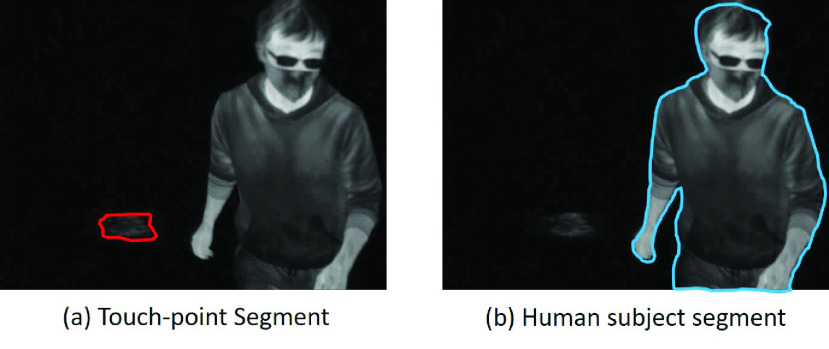
Manual labelled touch-point and human-subject.

The reference images were selected from the image sequences created by the 21 thermal videos. This set of images formulated a training and validation set for U-net evaluation, which was split with a ratio of 70/30 per standard practice [29]. A total of 90 images formed a basic dataset and 27 images were separately used for the validation. Although this was a relatively small dataset, these reference images encoded the features of touch-points and human-subjects well, and were manually labelled for semantic segmentation, as shown in Fig. 6. The labelling procedure required a precise pixel-wise segmentation by observing the difference of the pixel intensity in the image after Background subtraction.

#### 2) U-Net Training

**FIGURE 7.**
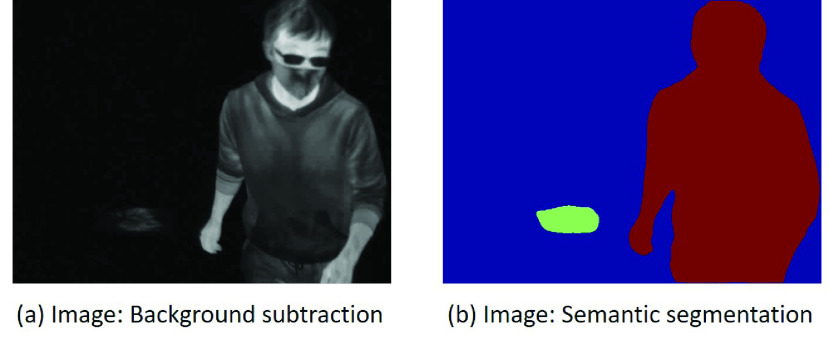
(a): The image after Background subtraction. (b): The result of semantic segmentation. Red segment denotes the human subject. Green segment denotes the touch-point. Blue segment denotes the background.

A generic U-net in [19] was implemented with Pytorch and trained on a GeForce RTX 2070 SUPER Graphics Cards. The cross-entropy was used as the loss function for multi-classes semantic segmentation [19]. The U-net was trained with 0.0005 learning rate and 40 epochs. The output of U-net is a tensor with shape $H \times W \times L$, where H and W represent the size of the image and L is the number of semantic labels. The labels for touch-point, human subject and the background can be acquired from the $3 \times 480 \times 640$ output tensor map in U-net [30]. The U-net model weights were selected at the epoch with the optimal touch-point prediction performance. An example of U-net segmentation is shown in Fig. 7.

#### 3) Cumulative Operation in Image Sequences

The output of $f_{Unet} (I_{i} - I_{0})$ is a semantic map where each pixel value is 0 (background), 1 (touch-point) and 2 (human-subject). Therefore, $\sum _{i}^{N} f_{Unet} (I_{i} - I_{0})$ can be defined as a summation of all the touch-point segments from each image. This cumulative operation can count the time of duration for the touch-point, i.e. how long a touch-point can last at a specific location.

### F. Evaluation Metric for Touch-Point Detection Pipeline

**FIGURE 8.**
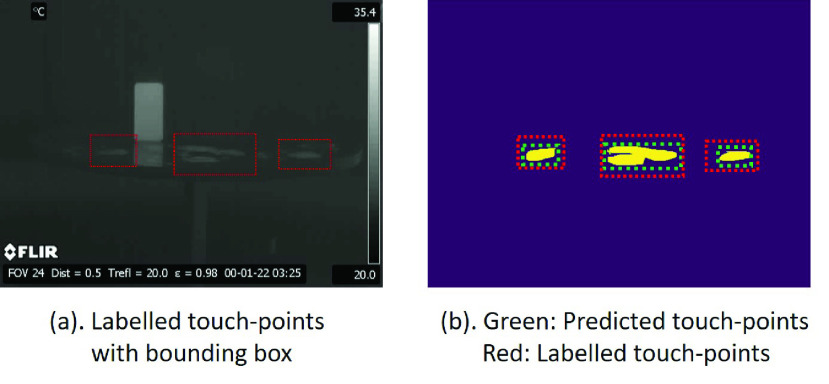
(a): Original image with touch-point bounding box labels. (b): Predicted (green) and labelled (red) touch-point regions.

Each frame in a video is processed through the touch-point detection pipeline and the output is an image mask that shows the regions of contact. To evaluate the pipeline, all the touch-points in each thermal video are labelled, as shown in Fig. 8 (a). It is noted that the labelling target is a “bounding box” instead of a segment, since the predicted result for the pipeline is a binary image with multiple small touch-point segments that can be grouped in a larger region. Instead of labelling the segments in the image, the “bounding box” can provide more effective information for cleaning and sanitation.

The predicted touch-point segments are usually not complete regions, and some touch-points are localized closely but not connected mutually. The effective touch-points are defined based on the following criteria:

#### 1) Remove Small Regions

**FIGURE 9.**
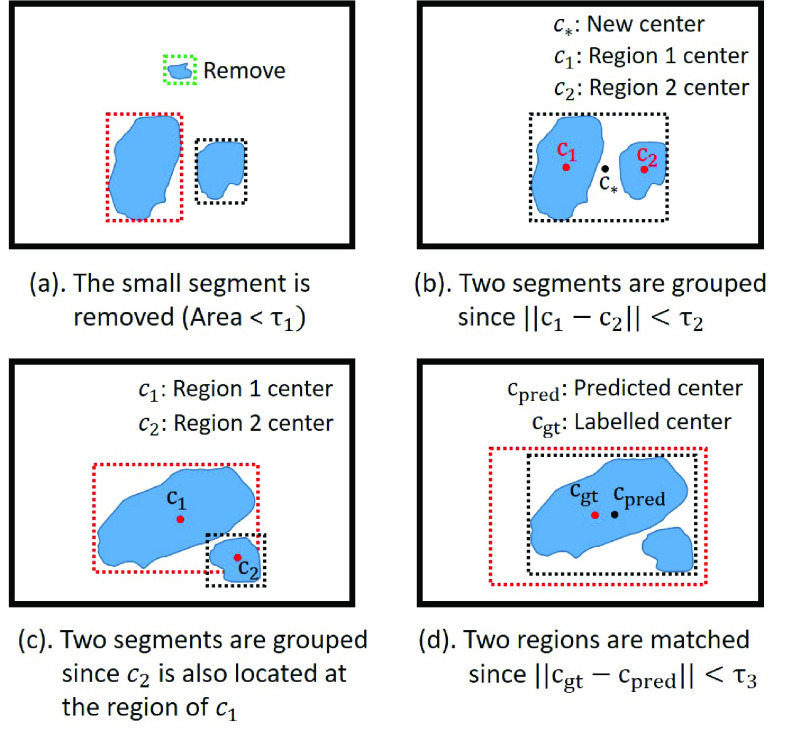
Criteria of effective touch-points.

The area of an effective touch-point should be greater than a threshold, in order to differentiate other small regions that are caused by incorrect U-net predictions or sensor noise. The region with area less a threshold $\tau _{1} = 100$ (unit: $pixel^{2}$) is removed from the list of effective touch-points (Fig. 9 (a)).

#### 2) Criteria of a Unified Cluster Region

The result of $I^{mask}$ includes a lot of small pixel regions that are classified as touch-points. However, most of these regions belong to the same touching region and this makes the grouping necessary. The centroid of each touch-point segment is computed by averaging the pixel coordinates inside the region. The regions with centroids less than the threshold $\tau _{2} = 30$ (unit: pixel) are grouped to formulate a new region. The new touch-point includes a rectangular area whose width and length are computed by the maximum and the minimum pixel coordinates within the region (Fig. 9 (b)).

It is likely that two bounding boxes on separate touch-points will overlap, since they are usually labelled to be slightly larger than the touch-points themselves. If one of the region centers is located at the region of another region, the two touch-points are grouped as one region (Fig. 9 (c)).

#### 3) Criteria of Successful Matching

Given a predicted region and a labelled region, with corresponding touch-point centroids calculated, a successful match is defined when the centroid distance between the two regions is smaller than a threshold $\tau _{3} = 30$ (unit: pixel), as shown in Fig. 9 (d).

## III. Experiments and Results

### A. U-Net Model Performance

The Dice similarity coefficient is used to evaluate the U-net performance for each epoch. Dice refers to two times the overlapping area divided by the total pixels of the two regions, which is measured between the predicted result and the ground truth [31]. A higher Dice coefficient indicates a better segmentation performance.

**FIGURE 10.**
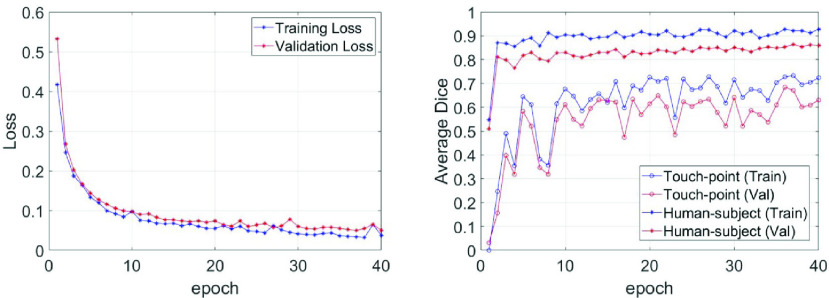
U-net model performance. (a): The training and validation loss of the U-net model. (b): The training and validation accuracy measured by average DICE coefficients. “val” denotes the validation.

Fig. 10 (a) illustrates the decrease of training and validation loss along epochs, which indicates the successfully training of the U-net model. Fig. 10 (b) illustrates the average Dice coefficient of the touch-point and human-subject calculated in training and validation datasets. The mean Dice for the validation data begins with 0.50 and increases to around 0.80 at the third epoch. The average Dice finally stabilizes at around 0.8 for the rest of the epochs. Even with a small training set, the U-net can quickly learn to segment the human-subject based on the features of the pixel intensity (relative to background) and the unique shape of the contour.

**FIGURE 11.**
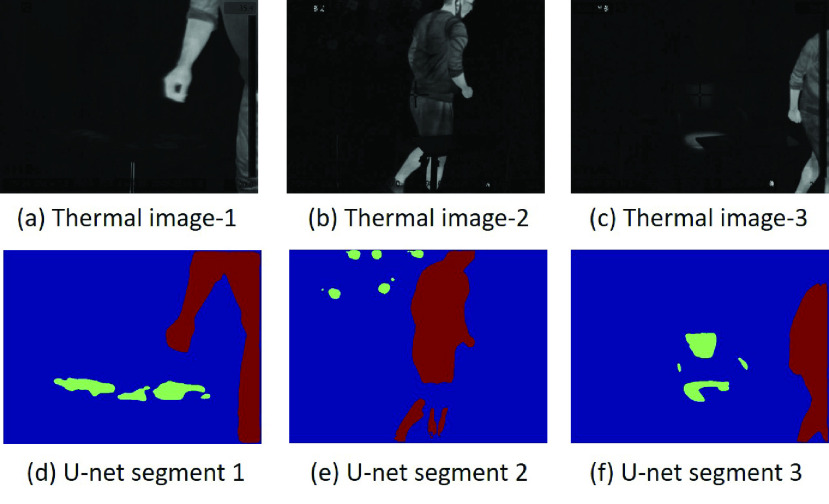
The result of U-net single image analysis. (a), (b) and (c) are the thermal images after Background subtraction. (d), (e) and (f) are U-net segmented images. Different colors indicate different semantic labels. Red: subject foreground, Green: Touch-point, Blue: Background.

The U-Net from the $36^{th}$ epoch was chosen for the touch-point detection pipeline because of the best validation performance. At this epoch, the average Dice coefficients for touch-point and human-subject prediction are 67.1% and 86.4%, respectively. Fig. 11 illustrates the examples of U-net segmenting touch-points and human-subjects in several frames of the thermal videos.

### B. Evaluation Results of the Touch-Point Detection Pipeline

For system testing, the touch-point detection pipeline is evaluated on both the training (21 videos) and testing sets (9 videos). The output of the detection pipeline is an image mask that shows the touch-point segments, which are grouped to formulate the rectangular regions. Each detected region is assigned with a label (1 or 0) as an indication of the touch-point detection. The ground truth information and the predicted results can be used to create a confusion matrix. Since the untouched regions cannot be labelled in this study, the definitions of True Positive (TP), True Negative (TN), False Negative (FN) and False Positive (FP) are slightly different from the generic definition of a classification model:

• TP (True positive): Successful match between the predicted and labelled touch-point.
• TN (True negative): Successful match between the predicted un-touch-point regions. This is not measured in this study as the untouched regions cannot be labelled. In fact, all the pixel regions that are not labelled can be considered as TNs and this should not be counted.
• FN (False negative): The labelled touch-point exists but not correctly predicted by the method. High FNs indicates a greater risk event as many touch-points are not predicted by the system.
• FP (False positive): The predicted touch-points exist but the labels does not. High FP ratio is an indication of many regions being classified as touch-points but actually not being touched.

**FIGURE 12.**
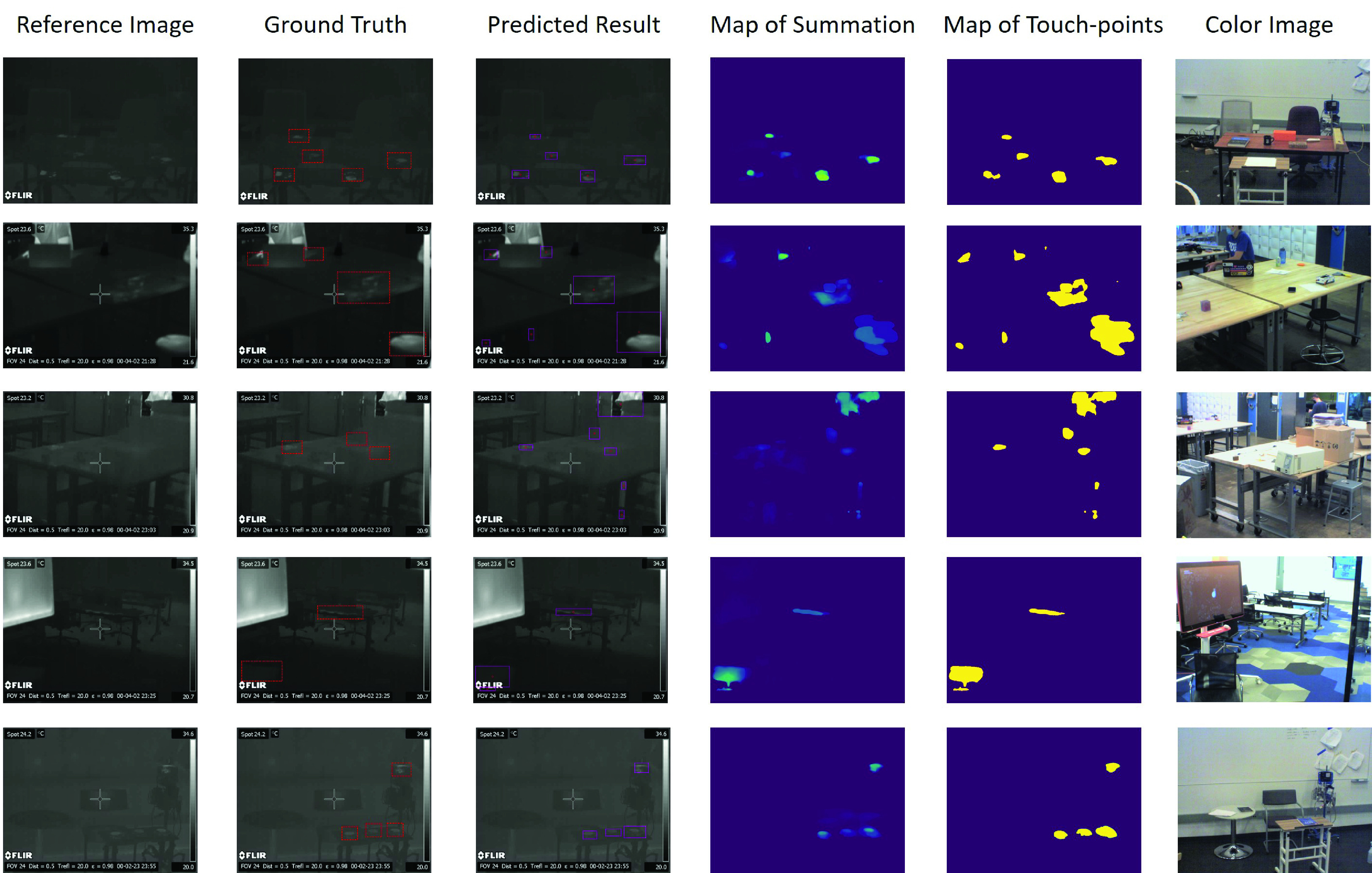
Five examples of touch-point detection in different scenes. Column 1: the reference thermal images with the touch-point features (only for visualization); Column 2: the ground truth of the touch-point regions; Column 3: the predicted touch-point regions; Column 4: the map of summation refers to the summation of the touch-point segments; Column 5: the binary mask of the summation map after filtering the small intensity pixels (low duration time); Column 6: the color image for visualization (the thermal image and the color image are not aligned). Particularly, row 2, 3 and 4 shows the special examples where some objects in the frame were moving during the video collection, which were also classified as the touch-points.

**FIGURE 13.**
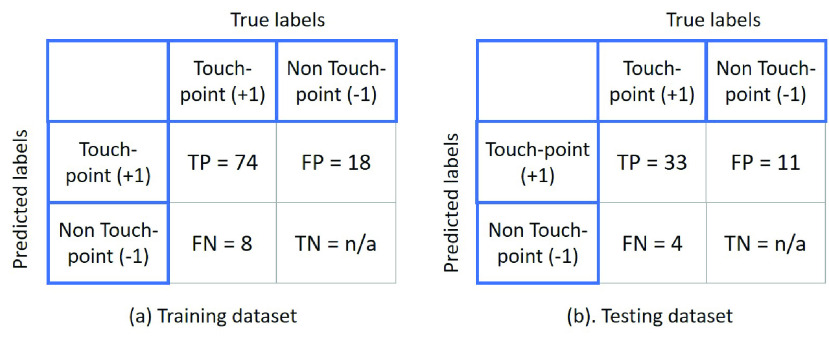
Confusion matrix of the touch-point detection model. The true negative (TN) is not reported since the cases of TN are the non-matched touch-points regions that cannot be counted for this study.

Fig. 13 illustrates the confusion matrix for the touch-point prediction. Since the use case for this system is for targeted cleaning, clearly FNs should be minimized and FPs are less of a problem. For both datasets, the TP is much higher than FP and FN, and FP is higher than FN. This indicates that the model can predict the touch-points with good precision and is more prone to FPs rather than FNs. In addition, the five example results in Fig. 12 also validate the conclusion that the proposed method tends to predict more touch-point regions that are actually not labelled, i.e. high False positive ratio.

Table. I summarizes the statistical measurements of the touch-point prediction model including the precision, F1-score, False negative rate (FNR) and False discovery rate (FDR). Precision refers to the percentage of the correct touch-points out of the total predicted cases. F1 score is a weighted average of precision and recall (True positive rate). These two measurements are commonly used to measure the effectiveness of our model.

**TABLE 1:** Statistical Measurements of the Touch-Point Prediction Model

	Training dataset	Testing dataset
Precision	80.4%	75.0%
F1-score	85.1%	81.5%
FNR	9.8%	10.8%
FDR	19.6%	25.0%

## IV. Discussion

For touch-point detection, the precision of 80.4% (training) and 75.0% (testing) indicate that the proposed system can robustly predict touch-point regions with a relatively good success rate. The achieved precision of the touch-point detection can demonstrate the feasibility of using thermal videos for touched regions tracking. In addition, the F1-scores of 85.1% (training) and 81.5% (testing) suggest a stability of the pipeline in predicting touch-point regions. It is noted that the False discovery rates (FDR) for training (19.6%) and testing (25.0%) are about twice as high as the false negative rates (FNR). This indicates that the proposed approach is more prone to false positive than false negative. This is preferable from a sanitation control standpoint, since the system can find the regions that matches the ground truth information, as well as more regions that are not labelled. A low ratio of false negative detection validates the feasibility of our system in touch-point detection, with a small probability of missing a touched region.

The U-net evaluation result shows that the model can achieve 67% average Dice coefficient for touch-point segmentation. This indicates that a U-net model with relatively low Dice coefficient can still achieve 75% precision for the testing experiments of the touch-point detection. High FPs and low FNs validates this founding because the U-net is prone to classifying all the moving objects and targets with high intensity changes as touch-points. Nevertheless, more FPs is much better than more FNs since this shows a lower probability of missing touch-point regions.

### A. Limitations

This study models the touch-point detection problem as a semantic segmentation task and only uses the spatial features (e.g shape of the segments) without explicit consideration of the temporal features, other than aggregating touch-points over the length of the video. This summation of all the resulted image mask from the single-frame analysis (Fig. 5. (a)) can only use part of the temporal features in the video and more features should be extracted based on the relations among consecutive images. Furthermore, modelling the thermal signature is another important problem, and studying the effects of temperatures among the contacted objects, material thermal property and the time of contact will be beneficial for developing a more accurate touch-point detection method.

Though deep learning provides powerful tools for computer vision problems, the physical modelling can be useful for designing robust feature extraction metrics and classifiers. The temporal features can be easily measured by the thermal videos and this can provide guidance for better machine learning approaches. The future work should focus on the temporal feature extraction and using machine learning tools to develop a robust touch-point classifier.

### B. Applications

#### 1) Application 1: Touch-Point Risk Assessment by the Time of Contact

**FIGURE 14.**
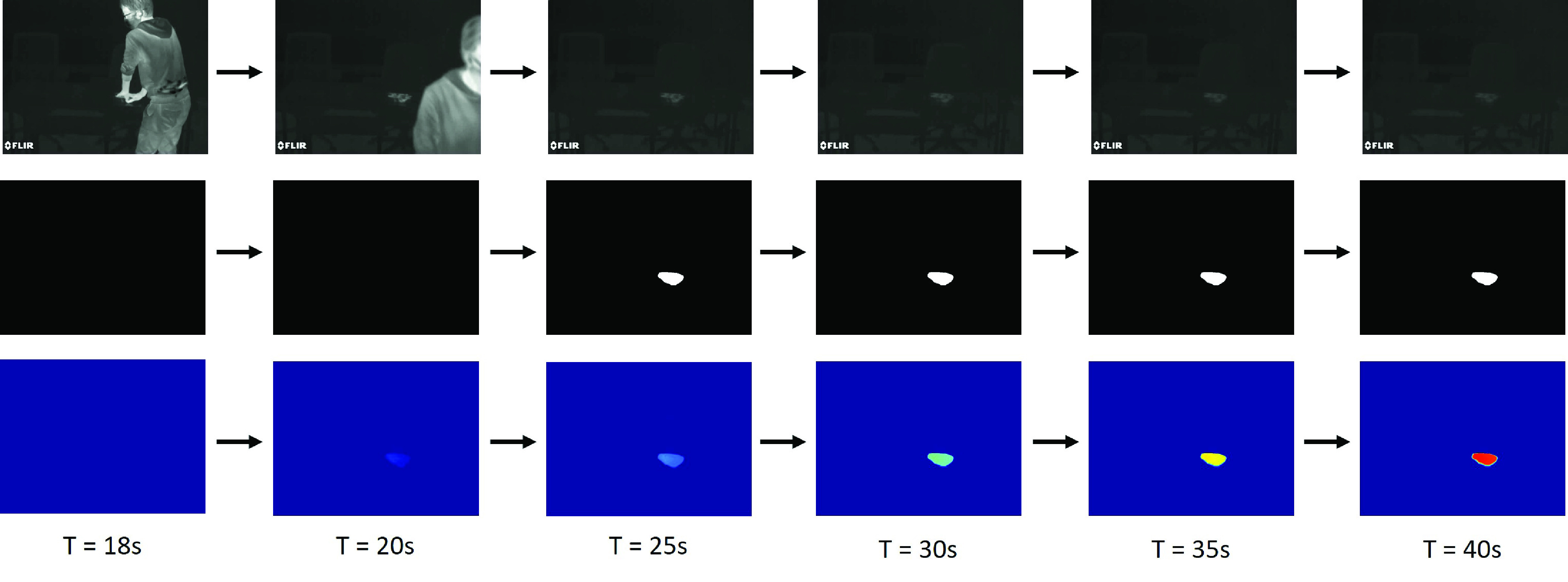
The changes of thermal images, segmented images and cumulative probability maps at different time steps. The first row shows the thermal image sequence. The touched surface shows higher pixel intensity compared with the non-contacted surfaces. The second row illustrates the segmented touch-points at each time step. The third row shows the cumulative probability map of the touch-point segments. The color intensity indicates the time of duration for the touch-point detection.

One important application is to count the time of duration for the touch-point, with longer time assumably correlating with high probability of infection transmission to or from the surface. For example, Fig. 14 shows three image sequences of the thermal images, the touch-point segments and the cumulative probability map of the touch-point. With increasing time, the confidence of the touch-point detection can be described by the cumulative probability map and the users can assess the risk of virus transmission by observing the color intensity.

#### 2) Application 2: Touch-Point Visualization

**FIGURE 15.**
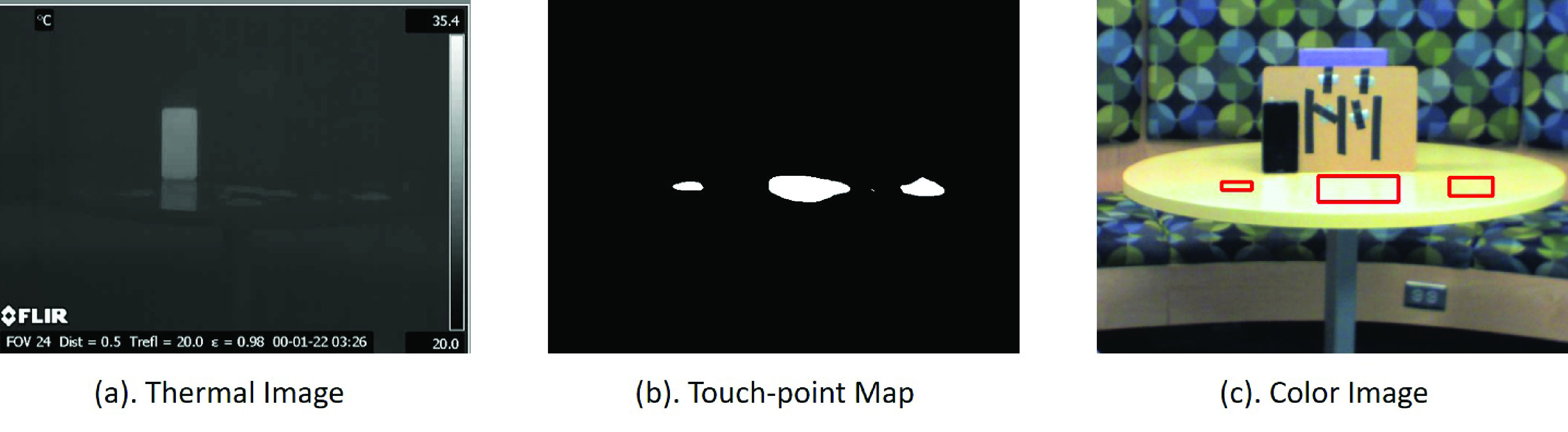
Thermal to color mapping after rough registration. (a): Raw thermal image, (b). Final mask showing touch-points. (c). Logged surface touch-points (red regions) aligned with the color image to delineate the cleaning targets.

Another useful application of the touch-point detection pipeline is to improve the efficiency of cleaning and sanitation in shared spaces. This technology can notify the cleaning staff of high-risk surfaces that are in need of cleaning, which can be achieved through calibration of the thermal camera and the color camera to create a thermal-to-color registration. For this to be user-friendly, the results of the touch-point regions can be displayed in a color image. For example in Fig. 15, the touch-points regions can be registered to the color image for visualization.

## V. Conclusion

This paper introduces a novel approach to detect the touched positions at contaminated surfaces in order to prevent indirect viral transmission. We propose a touch-point detection pipeline by using a thermal image sequence, which consists of a single-frame analysis of touch-point segmentation with the U-net deep learning framework, and a multi-frame analysis of cumulative touch-point segments. The experiments were conducted and evaluated from a new dataset of 30 thermal videos, with 21 for U-net training and validation, and 9 videos keeping separate for the test of the touch-point pipeline. The result shows a 75.0% precision and a 81.5% F1-score for the testing experiments of the touch-point detection. Future work includes improving the touch-point detection pipeline by considering the temporal features and collecting more data for model training. This study can show potential applications of preventing the indirect spreading of the viral and bacterial pathogens by detecting and reporting the touched surfaces.
